# Integration of CT and MRI imaging with plastination for enhanced veterinary anatomy education: a study on the crab-eating fox (*Cerdocyon thous*)

**DOI:** 10.3389/fvets.2025.1600763

**Published:** 2025-08-07

**Authors:** Mayla Magalhães de Oliveira Alcobaça, Athelson Stefanon Bittencourt, Yuri Favalessa Monteiro, Khallil Taverna Chaim, Aureo Banhos, Antônio Chaves de Assis Neto

**Affiliations:** ^1^Department of Surgery, Faculty of Veterinary Medicine and Animal Science, University of São Paulo, São Paulo, Brazil; ^2^Department of Morphology, Federal University of Espírito Santo, Vitória, Brazil; ^3^LIM44, Hospital das Clínicas of University of São Paulo Medical School, University of São Paulo, São Paulo, Brazil; ^4^Department of Biology, Federal University of Espírito Santo, Alegre, Brazil

**Keywords:** anatomical education, crab-eating fox, computed tomography, cross-section, magnetic resonance imaging, plastination, veterinary education, wild

## Abstract

**Introduction:**

With the advancements in diagnostic imaging and its increasing use in veterinary medicine, it is essential for professionals to continuously update their knowledge and seek training in the anatomy of various wild animal species. Plastination allows for the preservation of biological tissues and their anatomical details, enhancing the study and identification of structures.

**Methods:**

This study aimed (1) to describe in detail the anatomy of the thoracic and pelvic limbs of the crab-eating fox (*Cerdocyon thous*) using plastinated metameric sections correlated with computed tomography (CT) and magnetic resonance imaging (MRI), and (2) to analyze tissue shrinkage resulting from plastination. Limbs from four animals of both sexes were scanned using CT and MRI, and the resulting images were saved in DICOM format. The limbs were then plastinated using the silicone technique with forced impregnation at −18 °C, including fixation, dehydration, impregnation, and curing. Measurements of weight, thickness, and volume were taken before dehydration and after curing to assess shrinkage. Three-dimensional reconstructions and multiplanar virtual dissections were also performed to identify and describe anatomical structures.

**Results:**

The study enabled detailed identification and description of anatomical structures in plastinated transverse sections and their correlation with corresponding axial CT and MRI images, as well as the 3D reconstruction of the limbs for anatomical correspondence with each sectioned region. Qualitatively, the specimens showed excellent preservation of anatomical features, allowing for clear visualization and identification of structures while maintaining their syntopic relationships. The plastinated material was rigid, easy to handle, odorless, and free from toxic characteristics. Quantitative analysis demonstrated minimal shrinkage in the plastinated tissues.

**Discussion:**

Plastinated metameric sections of the thoracic and pelvic limbs of *Cerdocyon thous*, when correlated with CT and MRI, proved to be an effective tool for anatomical studies. This integrated approach improves anatomical understanding, supports the interpretation of imaging studies, enhances educational resources, and benefits veterinary professionals in both anatomy and diagnostic imaging. The minimal tissue shrinkage observed did not compromise the quality or anatomical integrity of the specimens.

## 1 Introduction

The crab-eating fox (*Cerdocyon thous*) is a wild canid in the order Carnivora (1). Its geographic distribution is extensive, and it is the most widespread species among neotropical wild canids (2). It has short limbs that facilitate its locomotion in forests (1). Because of human interference in their natural habitats, crab-eating foxes are often found near roads and highways, where many fall victims of vehicular accidents, leading to various injuries and traumas of varying severity (3).

With advancements in diagnostic imaging and increasing demand in clinical practice, anatomical knowledge has become indispensable. Professionals must constantly update their knowledge and undergo extensive training to confidently distinguish anatomical structures for accurate image interpretation, thereby enhancing their ability to apply this knowledge to clinical reports (4).

Computed tomography (CT) and magnetic resonance imaging (MRI) have gained significant importance in veterinary medicine. These techniques generate detailed images that allow precise evaluation of anatomical relationships without overlap. They also offer better resolution and higher structural contrast than the traditional methods (5, 6).

Plastination is an anatomical technique that replaces tissue fluids with a curable polymer, thereby increasing the durability of specimens while preserving their anatomical structures and components (7, 8). It serves as an excellent educational tool aiding anatomical identification and promoting deeper knowledge (7–9).

Based on the hypothesis that imaging techniques combined with plastination can assist in the interpretation and study of metameric sections of limbs, and that plastination produces reliable specimens with minimal shrinkage, the objective of this study was (1) to describe in detail the anatomy of the thoracic and pelvic limbs of the crab-eating fox (*C. thous*) using correlated plastinated metameric sections with CT and MRI; and (2) to analyze tissue shrinkage resulting from plastination.

## 2 Materials and methods

### 2.1 Animals

The project was approved by the Ethics Committee on the Use of Animals (CEUA) of the Faculty of Veterinary Medicine and Animal Science (FMVZ) at the University of São Paulo (USP), process 4143100220, and by the Federal University of Espírito Santo, process 31/2019, as well as by the Biodiversity Authorization and Information System (Sisbio), processes 31762-5, and 78227-3.

In this study, the pelvic and thoracic limbs of four roadkilled animals were used. The limbs were frozen shortly after the animals died and stored for up to a year. Before performing the procedures, the limbs were thawed at 7–10°C for 48 h.

### 2.2 Acquisition of imaging exams

CT and MRI scans were performed on the thoracic and pelvic limbs. The limbs were positioned in the lateral and ventral decubitus positions on the examination table in the most symmetrical manner, and scans were performed. The images were saved in the DICOM format for later analysis.

For CT, a Philips Brilliance device with 64 channels was used, calibrated at 120 kV and 332 mA with a 512 × 512 matrix. Two window settings were applied: for bones, window width (WW) of 4,095 and window level (WL) of 600; and for soft tissues, WW of 350 and WL of 70. The slice thickness was set to 1 mm.

For MRI, a SIEMENS 7 Tesla machine was used to acquire images with T2W sequences (TR: 500, TE: 86, matrix 640 × 320, WW 402, and WL 169). A coil was positioned over the limbs of the animal with a slice thicknesses of 0.6 mm.

### 2.3 Plastination technique

The thoracic and pelvic limbs of three animals were sectioned and subjected to the silicone plastination technique through forced impregnation at a low temperature (−25°C), following the method described by von Hagens, Tiedemann, and Kriz (7).

#### 2.3.1 Fixation

After thawing, the limbs were positioned, extended, and fixed in 10% formalin, infiltrating the musculature, and immersed in the solution for 4 weeks. After fixation, the limbs were cooled for 5 days at 5°C, then transferred to freezing at −25°C for 1 week.

Next, the limbs were embedded in polyurethane (PU) to facilitate and standardize the sections by adapting Monteiro et al. (10) method for use in animals. Equal parts of polyurethane components “A” and “B” were mixed and poured into a custom-made box containing the frozen limbs, ensuring complete immersion in the PU compound. The limbs were frozen at −25°C for 7 days.

After freezing, a band saw (Skymsen, SSI model No. 1974) was used to section the limbs into 13 mm thick cross sections. The sections were labeled to preserve anatomical positioning.

#### 2.3.2 Dehydration

A 10-volume hydrogen peroxide solution (3%) was used to whiten the slices by immersion at room temperature for 24 h. Subsequently, the hydrogen peroxide solution was removed by immersing the slices in running water for 12 h. Dehydration was then carried out in 4-weekly immersion baths in pure acetone (100%) at −25°C. After the fourth bath, the acetone purity was measured with an acetonometer, with concentrations exceeding 99% (v/v).

#### 2.3.3 Impregnation

The dehydrated slices underwent forced impregnation at −18°C, being immersed in a mixture composed of low-viscosity silicone (Poliplast 1 - P1; Polisil®, Brazil), polydimethylsiloxane (PDMS), and the catalyst dibutyltin dilaurate (DBTL), in a 100:1 (m/m) ratio. This process was conducted in a vacuum chamber for 24 h. Vacuum pressure was applied gradually at two bubbles per second at a fixed observation point (11). Vacuum progression was monitored by measuring mercury levels and using a digital manometer. Once bubble formation ceased, the maximum vacuum was applied to complete the process. The impregnation lasted 28 days, achieving a minimum pressure (maximum vacuum) of 6 mmHg.

#### 2.3.4 Curing

The slices were drained at room temperature (20–25°C) for 4 weeks and then placed in a curing chamber, where tetraethyl orthosilicate (TEOS) was volatilized to promote crosslinking between molecules, solidifying the samples. Curing was considered complete after 2 days of volatilization.

### 2.4 Statistical measurement and analysis

To analyze shrinkage in the plastinated specimens, weight (g) was measured on a calibrated scale, volume (mL) in graduated vats, and thickness (cm) using an analog caliper at the four edges of the sections (mean values of the four extremities were used). Measurements were performed at two time points: t1 (before dehydration) and t2 (after curing). The arithmetic mean of each variable was calculated. The difference in means between t1 and t2 was calculated with a 95% confidence interval.

Prior to applying the paired Student’s t-test, data normality was assessed using the Shapiro–Wilk W test. A paired Student’s t-test was applied to the samples, reporting one-tailed *p*-values to test for shrinkage, where Ha: t1 > t2, indicating that the difference between t1 and t2 was greater than zero for volume, weight, and thickness. Statistical significance was set at *p* < 0.05. Additionally, percentage reduction for each sample was calculated as (t2 − t1) ÷ t1 × 100, and the mean reduction and standard deviation were reported.

### 2.5 Photo documentation and image processing

The proximal surface of each segment was photographed using a Canon EOS Rebel T3i digital camera with an EF-S 18–135 mm lens. The images were correlated and identified with CT and MRI scans using the RadiAnt DICOM Viewer (12) and 3D Slicer (13) software. These programs have been employed for multiplanar reconstruction, 3D rendering, filtering, and segmentation of structures. Finally, virtual dissection and identification of the anatomical structures in each correlated image were conducted according to Veterinary Anatomical Nomina (14). All images were presented in a proximal view, with the left side of the image corresponding to the viewer’s left side for easier identification.

## 3 Results

At the end of plastination, sections were obtained, arranging them corresponding to the segments of the thoracic and pelvic limbs of the crab-eating fox in a proximo-distal order. The results demonstrated that the sectioned slices were easy to handle and durable without detaching from the specimens. Additionally, the preserved rigidity of the anatomical components allows for direct handling without breaking or detachment.

The segmented slices exhibited a uniform color, with no signs of degradation or tissue alteration. The staining of the metamers remained consistent and varied according to the characteristics of the structures (muscle, tendon, bone, and vessels). These variations were influenced by the features of the specimen and the bleaching protocol used in plastination. However, the morphometry may vary according to the individual characteristics of each structure, considering the different levels of tissue retraction.

No significant anatomical variations were observed among the analyzed specimens. Additionally, this species does not exhibit marked sexual dimorphism that could interfere with the anatomical parameters evaluated, ensuring consistency in the data across male and female individuals.

The olfactory characteristics of the odors were similar to those of the post-cured silicone used in the technique. The visual aspects remained anatomically comparable to those of the specimens before fixation. The anatomical structures were well-preserved, showing no alterations and allowing for easy identification. The sliced limb arrangements are presented in a proximal-to-distal direction. Visualization, 3D rendering, multiplanar reconstruction, segmentation, and virtual dissection of the limbs were performed using CT and MRI images. Through software analysis, it was possible to correlate anatomical structures, identify them, and segment the areas of interest. While handling the material, it was possible to cut and exclude structures, differentiate the stratigraphy of tissues, and achieve greater accuracy in anatomical descriptions.

### 3.1 Aspects of tissue retraction

Ninety-three sections corresponding to the plastinated anatomical regions of the thoracic and pelvic limbs were examined. Table 1 shows an average reduction of 3.20 g and 2.59 g, respectively, with weight reductions of 29.80 and 25.12% in the right and left limbs, respectively. The average reduction for the right and left pelvic limbs was 3.40 g (23.53%) and 3.04 g (23.89%), respectively. The *p*-value was less than 0.001 for all four limbs (Table 1).

**Table 1:** Degree of tissue retraction relative to the weight of plastinated metamers in the limbs of the crab-eating fox, presented as total limbs and as thoracic and pelvic limbs, subdivided into right and left antimeres.

Anatomical regions	*n*	Weight (g)		Shrinkage (g)					
		Before dehydration	After curing	Mean	Lower 95% CI	Upper 95% CI	*p*-value	Shrink %	SD%
Right thoracic limb	22	10.99	7.79	3.20	2.36	4.04	<0.001	−29.80%	−29.80%
Left thoracic limb	20	10.32	7.73	2.59	1.86	3.32	<0.001	−25.12%	4.86%
Thoracic limbs	**42**	**10.67**	**7.76**	**2.91**	**2.36**	**3.46**	**<0.001**	**−27.57%**	**5.21%**
Right pelvic limb	25	12.92	9.52	2.91	2.36	3.46	<0.001	−27.57%	5.21%
Left pelvic limb	26	12.35	9.31	3.40	1.96	4.84	<0.001	−23.53%	8.03%
Pelvic Limbs	**51**	**12.63**	**9.41**	**3.22**	**2.34**	**4.09**	**<0.001**	**−23.71%**	**7.14%**
Total limbs	**93**	**11.74**	**8.67**	**3.08**	**2.55**	**3.61**	**<0.001**	**−25.46%**	**6.60%**

SD: Standard deviation, *n*: Number of observations, CI: Confidence interval, g: Grams.The values in bold represent the resulting combination of the sums of all sections of the thoracic limbs, pelvic limbs, and the total limbs, respectively.

**Figure 1:**
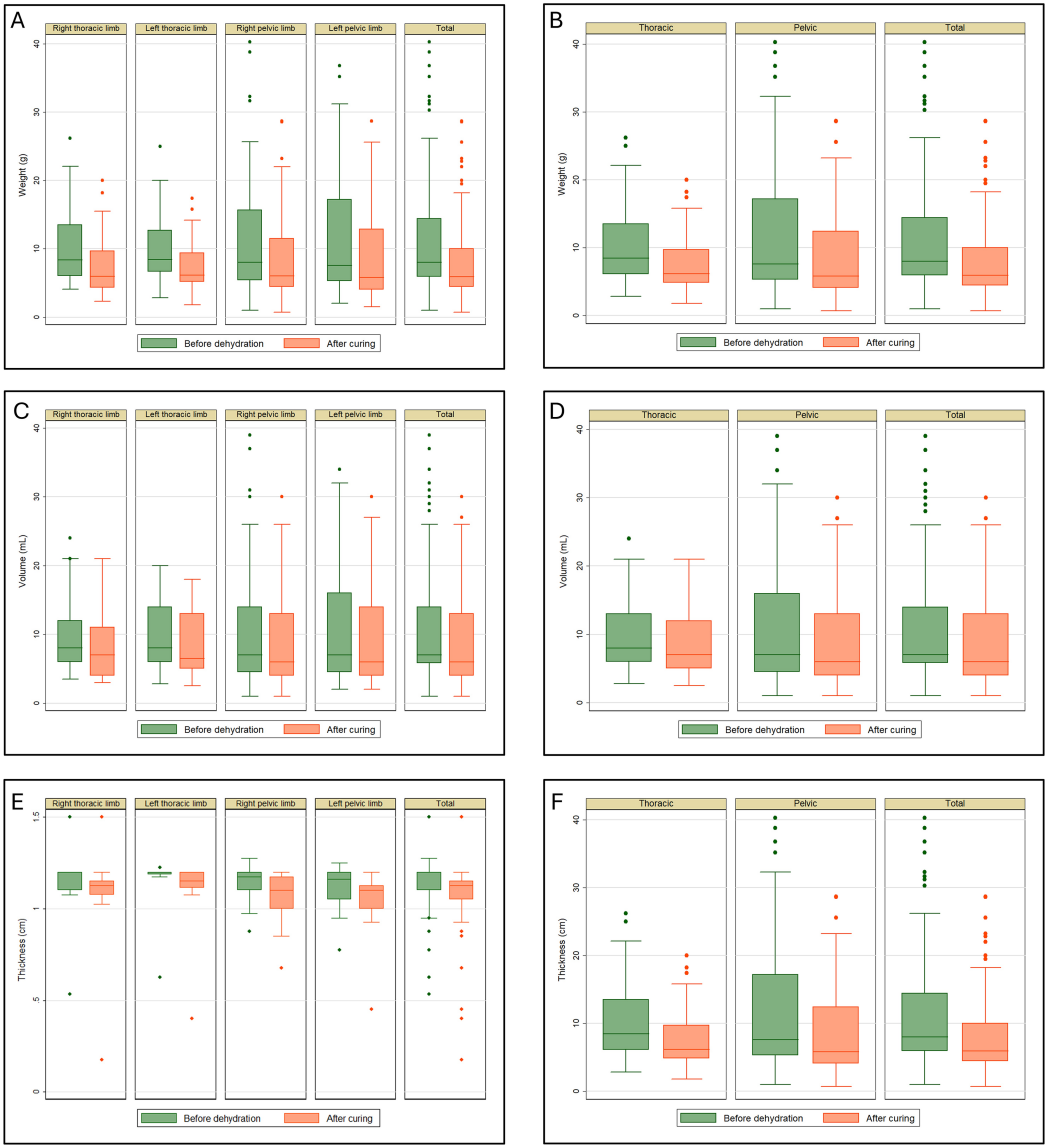
Tissue retraction in plastinated metamers of the thoracic and pelvic limbs of the crab-eating fox. **(A)** Weight (g), divided into right and left antimeres; **(B)** total weight (g) of the thoracic and pelvic limbs; **(C)** volume (mL), divided into right and left antimeres; **(D)** total volume (mL) of the thoracic and pelvic limbs; **(E)** thickness (cm), divided into right and left antimeres; **(F)** total thickness (cm) of the thoracic and pelvic limbs.

The data distribution in Figure 1A shows the maximum and minimum values for the anatomical regions, with the minimum being greater than zero and the maximum exceeding 30 g identified in the left pelvic limb. Outliers appear in all charts but are only present at the upper limit. The median shows variations but remains close to the first quartile in all analyzed regions (Figure 1A).

The weight reduction in the thoracic and pelvic section showed an average retention of 2.91 g (27.53%) and 3.22 g (23.71%), respectively. The combined average reduction for all thoracic and pelvic limb sections was 3.08 g, representing a total weight reduction of 25.41%. The *p*-value was less than 0.001 for the analysis of weight retraction of all thoracic and pelvic limb sections (Table 1). Figure 1B shows the maximum and minimum values for the anatomical regions, with a minimum greater than zero and a maximum exceeding 30 g identified in the pelvic limb. Outliers appear in all charts but are only present at the upper limit. The median shows variations but remains close to the first quartile in all analyzed regions.

The retraction volume in milliliters for the right and left antimeres showed a mean retention of 1.41 mL (14.72%) and 1.13 mL (12.80%), respectively. The average volume reduction for the right and left limbs was 2.37 mL (19.08%) and 1.60 mL (13.85%), respectively. The *p*-value was less than 0.001 for all four limbs (Table 2). Figure 1C shows the maximum and minimum values for the anatomical regions, with a minimum greater than zero and a maximum exceeding 30 mL identified in the left pelvic limb. Outliers appeared in the charts for the right thoracic and pelvic limbs, as well as the grand total but only at the upper limit. The median shows variations but remains close to the first quartile in all analyzed regions.

**Table 2:** Degree of tissue retraction relative to the volume of plastinated metamers in the limbs of the crab-eating fox, presented as total limbs and as thoracic and pelvic limbs, subdivided into right and left antimeres.

Anatomical regions	*n*	Volume (mL)		Shrinkage (mL)					
		Before dehydration	After curing	Mean	Lower 95% CI	Upper 95% CI	*p*-value	Shrink %	SD%
Right thoracic limb	22	10.05	8.64	1.41	0.98	1.84	<0.001	−14.72%	9.00%
Left thoracic limb	20	9.60	8.48	1.13	0.84	1.41	<0.001	−12.80%	5.33%
Thoracic limbs	**42**	**9.84**	**8.56**	**1.28**	**1.02**	**1.53**	**<0.001**	**−13.80%**	**7.46%**
Right pelvic limb	25	12.23	9.86	2.37	1.34	3.39	<0.001	−19.08%	8.82%
Left pelvic limb	26	11.46	9.87	1.60	0.99	2.20	<0.001	−13.85%	7.00%
Pelvic Limbs	**51**	**11.84**	**9.86**	**1.97**	**1.40**	**2.55**	**<0.001**	**−16.42%**	**8.29%**
Limbs	**93**	**10.93**	**9.27**	**1.66**	**1.32**	**2.00**	**<0.001**	**−15.24%**	**7.99%**

SD: Standard deviation, *n*: Number of observations, CI: Confidence interval, mL: Milliliters.The values in bold represent the resulting combination of the sums of all sections of the thoracic limbs, pelvic limbs, and the total limbs, respectively.

Table 2 shows the retraction volume of the thoracic and pelvic limbs in milliliters, corresponding to 1.28 mL (13.80%) and 1.97 mL (16.42%), respectively. The average reduction for all limbs was 1.66 mL (15.24%). The *p*-value was less than 0.001 for all analyses (Table 2).

Figure 1D shows the maximum and minimum values for the anatomical regions, with a minimum greater than zero and a maximum exceeding 30 mL identified in the pelvic limb. Outliers appeared in the charts but only at the maximum limit, except for the thoracic limb post-cure analysis, where no outliers were present. The median shows variations but remains close to the first quartile in all analyzed regions.

Table 3 shows the reduction in thickness of the thoracic limb for both right and left antimeres, with a mean reduction of 0.06 cm (6.40%) and 0.05 cm (5.14%), respectively. The mean reduction for the right and left pelvic limbs was 0.06 cm (5.34%) and 0.07 cm (6.93%), respectively. The *p*-values were 0.002 for the right thoracic limb, 0.000 for the left thoracic limb, and less than 0.001 for the right and left pelvic limbs (Table 3).

**Table 3:** Degree of tissue retraction relative to the thickness of plastinated metamers in the limbs of the crab-eating fox, presented as total limbs and as thoracic and pelvic limbs, subdivided into right and left antimeres.

Anatomical regions	*n*	Thickness (cm)		Shrinkage (cm)					
		Before dehydration	After curing	Mean	Lower 95% CI	Upper 95% CI	*p*-value	Shrink %	SD%
Right thoracic limb	22	1.15	1.10	0.06	0.02	0.09	0.002	−6.40%	14.01%
Left thoracic limb	20	1.17	1.12	0.05	0.03	0.08	0.000	−5.14%	7.84%
Thoracic limbs	**42**	**1.16**	**1.11**	**0.05**	**0.03**	**0.07**	**<0.001**	**−5.80%**	**11.38%**
Right pelvic limb	25	1.14	1.08	0.06	0.04	0.08	<0.001	−5.34%	5.56%
Left pelvic limb	26	1.11	1.10	0.01	−0.07	0.09	0.423	−0.75%	−0.75%
Pelvic Limbs	**51**	**1.12**	**1.09**	**0.03**	**−0.01**	**0.07**	**0.060**	**−3.00%**	**14.26%**
Limbs	**93**	**1.14**	**1.10**	**0.04**	**0.02**	**0.07**	**0.001**	**−4.27%**	**13.05%**

SD: Standard deviation, *n*: Number of observations, CI: Confidence interval, cm: Centimeters.The values in bold represent the resulting combination of the sums of all sections of the thoracic limbs, pelvic limbs, and the total limbs, respectively.

The data distribution in Figure 1E shows the maximum and minimum values for the anatomical regions, with a minimum >0.5 cm for both pelvic limbs and the total for all limbs and a minimum greater than 1.0 cm for both thoracic limbs. The maximum exceeded 1.0 cm in all the analyses. Outliers appeared in all chart analyses. The median shows variations but is generally close to the third quartile in the analyses (Figure 1E).

The reduction in thickness of the thoracic and pelvic limb sections showed an average reduction of 0.05 cm (5.80%) and 0.07 cm (6.15%), respectively. The average reduction for all limbs was 0.06 cm (5.99%). The *p*-value was less than 0.001 for all analyses (Table 3). Figure 1F shows the maximum and minimum values for the anatomical regions, with minimum greater than 1.0 cm for the thoracic limb and 0.5 cm for the pelvic limb. For all limbs, the maximum exceeded 1.0 cm in all analyses except during the post-healing period for the thoracic limb. Outliers appeared in all the Figures. The median shows variations across all the analyzed regions.

### 3.2 Sectional anatomy of the limbs

**Figure 2:**
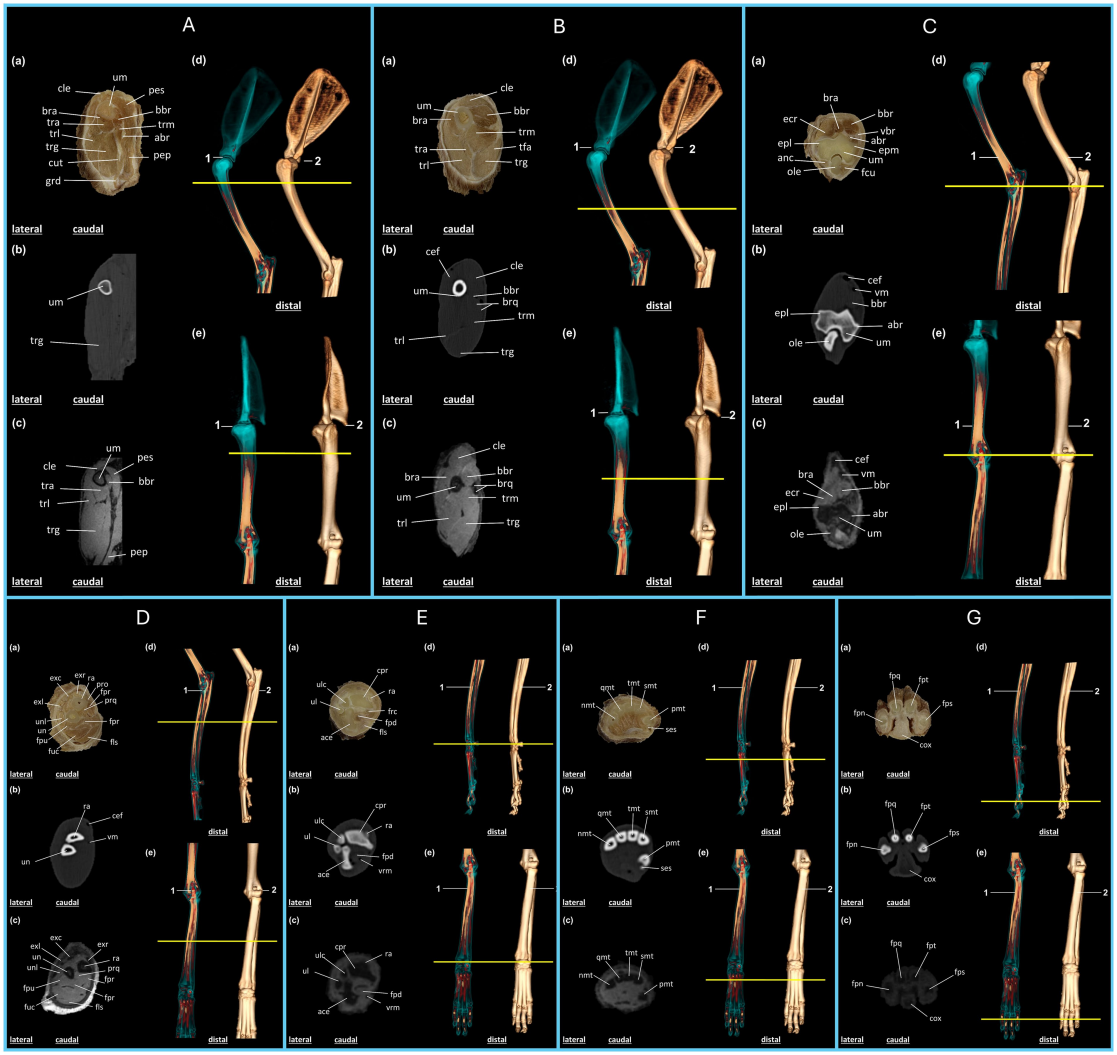
Anatomical regions of the thoracic limb of the crab-eating fox (*C. thous*) **(A–G)**: **(A,B)** Brachial region; **(C,D)** antebrachial (elbow) region; **(E–G)** hand region. Each anatomical region includes: (a) plastinated cross-section, (b) axial computed tomography section with bone window, (c) axial T2-weighted magnetic resonance imaging section. Three-dimensional reconstructions: (d) lateral view and (e) cranial view, highlighting the articular regions (1) and the skeleton (2), with a yellow line marking the section of the specimen limb. **(A)** abr: brachial artery; bbr: biceps brachii muscle; bra: brachialis muscle; cle: cleidobrachialis muscle; cut: cutaneous muscle of the trunk; grd: large dorsal muscle; pep: deep pectoral muscle; pes: superficial pectoral muscle; tra: triceps brachii muscle (accessory head); trg: triceps brachii muscle (long head); trl: triceps brachii muscle (lateral head); tmr: triceps brachii muscle (medial head); um: humerus. **(B)** bbr: biceps brachii muscle; bra: brachialis muscle; brq: brachial artery and vein; cef: cephalic vein; cle: cleidobrachialis muscle; tfa: tensor fasciae antebrachii muscle; tra: triceps brachii muscle (accessory head); trg: triceps brachii muscle (long head); trl: triceps brachii muscle (lateral head); trm: triceps brachii muscle (medial head); um: humerus. **(C)** abr: brachial artery; anc: anconeus muscle; bbr: biceps brachii muscle; bra: brachialis muscle; cef: cephalic vein; ecr: extensor carpi radialis muscle; epl: lateral epicondyle of the humerus; epm: medial epicondyle of the humerus; fcu: flexor carpi ulnaris muscle; ole: olecranon; um: humerus; vbr: brachial vein; vm: median vein. **(D)** cef: cephalic vein; exc: m. extensor digitorum communis; exl: m. lateral extensor digitorum; exr: m. extensor carpi radialis; fls: m. flexor digitorum superficialis; fpr: m. flexor digitorum profundus (radial head); fph: m. flexor digitorum profundus (humeral head); fpu: m. flexor digitorum profundus (ulnar head); fuc: m. flexor carpi ulnaris (humeral head); pro: m. pronator teres; prq: m. pronator quadratus; ra: radius; un: ulna; unl: m. ulnaris lateralis; vm: median vein. **(E)** ace: carpal attachment; cpr: radial carpus; fls: m. flexor digitorum superficialis; fpd: m. flexor digitorum profundus; frc: m. flexor carpi radialis; ra: radius; ul: ulna; ulc: ulnar carpus; vmr: radial median vein. **(F)** pmt: first metacarpal; smt: second metacarpal; tmt: third metacarpal; qmt: fourth metacarpal; nmt: fifth metacarpal; ses: sesamoid bone. **(G)** fps: proximal phalanx of the second digit; fpt: proximal phalanx of the third digit; fpq: middle phalanx of the fourth digit; fpn: middle phalanx of the fifth digit; cox: metacarpal pad.

**Figure 3:**
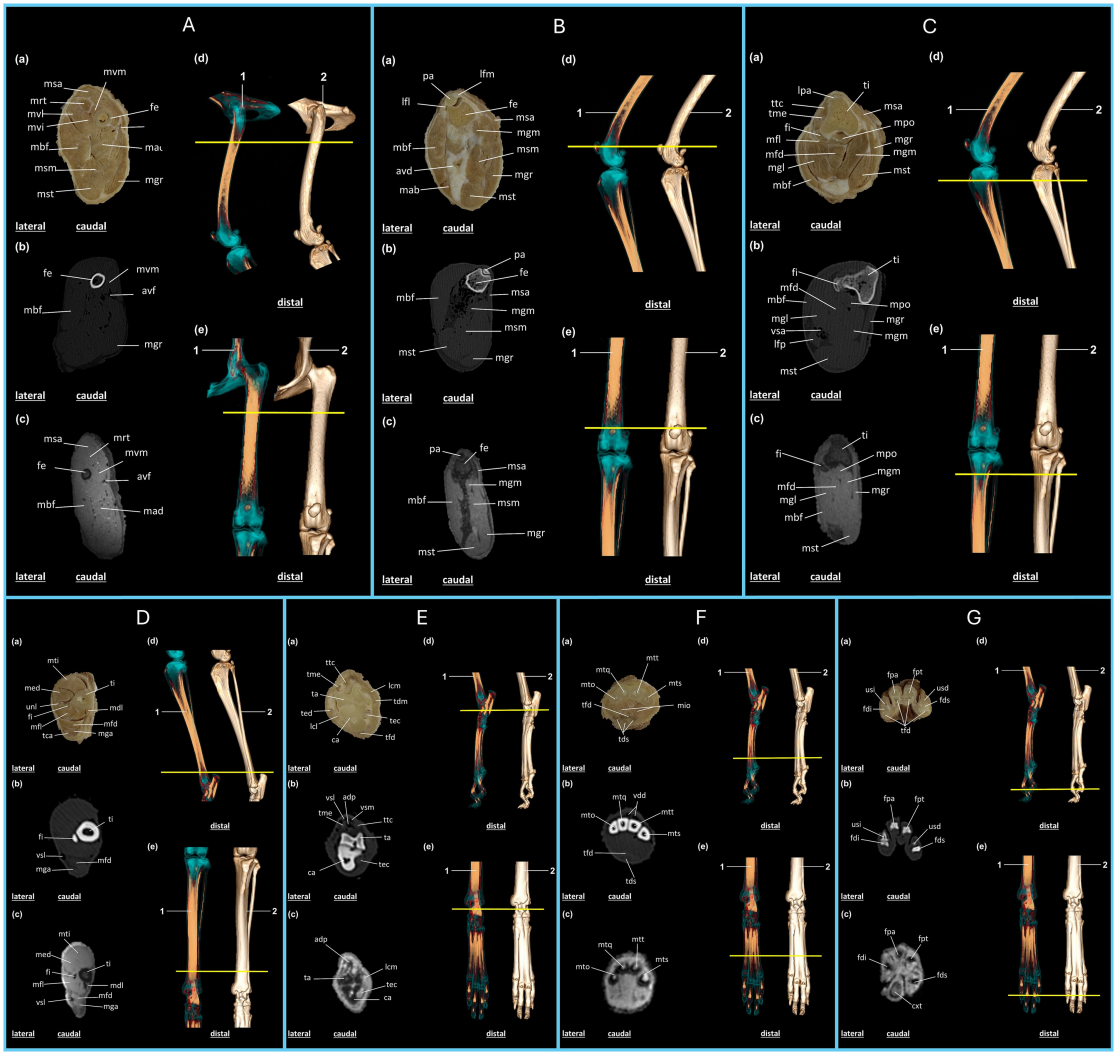
Anatomical regions of the pelvic limb of the crab-eating fox (*C. thous*) **(A–G)**: **(A)**, proximal femoral region; **(B)**, distal femoral region (knee joint); **(C)**, proximal region of the leg; **(D)**, distal region of the leg; **(E)**, tarsal region of the foot; **(F)**, metatarsal region of the foot; **(G)**, phalangeal region of the foot. Each anatomical region includes: (a) plastinated cross-section, (b) axial computed tomography section with bone window, (c) axial T2-weighted magnetic resonance imaging section. Three-dimensional reconstructions: (d) lateral view and (e) dorsal view, highlighting the articular regions (1) and the skeleton (2), with a yellow line marking the section of the specimen limb. **(A)** avf: femoral artery and vein; fe: femur; mad: m. adductor; mbf: m. biceps femoris; mgr: m. gracilis; mrt: m. rectus femoris; msa: m. sartorius; msm: m. semimembranosus; mst: m. semitendinosus; mvi: m. vastus intermedius; mvl: m. vastus lateralis; mvm: m. vastus medialis. **(B)** avd: distal femoral caudal artery and veins; fe: femur; lfl: lateral patellofemoral ligament; lfm: medial patellofemoral ligament; mab: m. abductor crural caudal; mbf: m. biceps femoris; mgm: m. gastrocnemius, medial head; mgr: m. gracilis; msa: m. sartorius; msm: m. semimembranosus; mst: m. semitendinosus; pa: patella. **(C)** fi: fibula; lfp: popliteal lymph node; lpa: patellar ligament; mbf: m. biceps femoris; mfd: m. superficial digital flexor; mfl: m. long digital flexor, first digit; mgl: m. gastrocnemius, lateral head; mgm: m. gastrocnemius, medial head; mgr: m. gracilis; mpo: m. popliteus; msa: m. sartorius; mst: m. semitendinosus; ti: tibia; tme: extensor digitorum longus tendon; ttc: cranial tibialis muscle; vsa: saphenous vein. **(D)** fi: fibula; mdl: m. long digital flexor; med: m. long digital extensor; mfd: m. superficial digital flexor; mfl: m. long digital flexor, first digit; mga: m. gastrocnemius; mti: m. cranial tibialis; tca: calcaneal tendon; ti: tibia; unl: peroneus longus muscle; vsl: lateral saphenous vein. **(E)** adp: dorsal artery of the foot; ca: calcaneus; lcl: lateral collateral ligament; lcm: medial collateral ligament; mfd: m. superficial digital flexor; ta: talus; tdm: m. medial digital flexor; tec: m. lateral digital flexor; ted: m. lateral digital extensor; tfd: m. deep digital flexor; me: m. long digital extensor; ttc: cranial tibialis muscle; vsl: lateral saphenous vein, cranial branch; vsm: medial saphenous vein, cranial branch. **(F)** mio: m. interosseous; mto: fifth metatarsal; mtq: fourth metatarsal; mts: second metatarsal; mtt: third metatarsal; tds: m. superficial digital flexor; tfd: m. deep digital flexor; vdd: common dorsal digital vein. **(G)** cxt: metatarsal pad; fdi: middle phalanx of the fifth digit; fds: distal phalanx of the second digit; fpa: middle phalanx of the fourth digit; fpe: proximal phalanx of the third digit; tfd: m. deep digital flexor; usd: ungual process of the distal phalanx of the second digit; usi: ungual process of the distal phalanx of the fifth digit.

The anatomical structures can be identified by correlating plastination techniques with the images obtained from the proximal surface of the plastinated sections and the corresponding CT and MRI scans (Figures 2, 3). Three-dimensional reconstruction from volumetric CT scans enabled the identification of anatomical structures and provided a three-dimensional view of the thoracic and pelvic limbs as well as the animal’s skeleton (Supplementary Videos 1, 2), with the articular regions highlighted in blue (Supplementary Videos 3, 4).

The brachialis and triceps brachii muscles were observed in the proximal region of the arm (Figure 2A), including their long, medial, lateral, and accessory heads. Because this is the most proximal region of the limb, the superficial and deep pectoral muscles, cutaneous muscle of the trunk, and large dorsal muscle are also visible in the plastinated section as well as in the CT and MRI scans. The proximal third of the humerus was visible near the humeral neck region. Owing to the varying tissue densities on the CT and MRI images and in the plastinated metamer, muscle surfaces were clearly delineated, facilitating the distinction of each structure in this region (Figure 2A).

The accessory, long, lateral, and medial heads of the triceps brachii extend into the distal third of the arm. In this region, the biceps brachii muscle exhibits a distinctive morphology, making it easily identifiable in the plastinated specimen as well as in CT and MRI scans. The brachialis muscle, which wraps around the humerus, also appears larger than that described in the previous section, and is easily distinguished using imaging techniques. Regarding vascularization, major vessels, such as the cephalic vein, brachial artery, and vein, were clearly identifiable in all imaging modalities (Figure 2B).

In the forearm, the biceps brachii muscle was identified in the plastinated specimen as well as in the CT and MRI scans, positioned lateral to the brachialis muscle, followed by the extensor carpi radialis muscle. The humeral condyle is distinctly visible along the lateral and medial epicondyles at the apex. The olecranon lies posterior to the humerus, with muscles such as the anconeus muscle laterally and the flexor carpi ulnaris muscle medially. The vascular structures were also well visualized, with the cephalic vein appearing superficial and cranial, whereas the median vein was observed in the medio caudal region (Figure 2C).

In the proximal third of the forearm, the extensor muscles of the fingers are positioned dorsally and dorsolaterally, with the extensor carpi radialis being particularly prominent. Flexor muscles, including the deep and superficial flexors of the fingers, are also evident in this region and are easily differentiated on MRI and plastinated sections. The radius and ulna support the leverage of the forearm and are identifiable on CT, MRI, and plastinated sections (Figures 2D–E).

In the proximal third of the hand, the carpal bones articulated closely with the radial and ulnar bones and distally with the metacarpals. The accessory carpal bones were arranged caudally, while the radial carpal bones were arranged cranially. The superficial and deep flexor muscles of the fingers are located lateral to the accessory carpal bone. Muscle structures were well identified in the plastinated specimen and on MRI. However, on CT, the bone and vascular aspects were more evident (Figure 2E).

The metacarpal bones were arranged proximally and distally, with the second to fourth metacarpals aligned mediolaterally. The first metacarpal was located medial and caudal to the other bones. The sesamoid bone was positioned adjacent and caudal to the first metacarpal and was identifiable on CT images (Figure 2F).

In distal sections of the hand, the proximal phalanges of the second, third, fourth, and fifth digits were visible, with the metacarpal pad located caudally in the palmar region. The visualization and identification of these structures were enhanced by the correlation of the three imaging techniques, each offering complementary perspectives.

The pelvic limb begins with the cranial sartorius muscle, followed by the quadriceps femoris, which includes the vastus lateralis, medialis, intermedius, and rectus femoris muscles. The femur is the sole bony structure supporting this section of the limb, whereas the femoral artery and vein provide caudomedial vascularization. The adductor muscle is located caudal and lateral to the biceps femoris, whereas the semimembranosus, semitendinosus, and gracilis muscles occupy the caudal region (Figure 3A).

In plastinated sections, on CT and MRI, the patella articulates with the femur, and its contours are well-defined and clearly identified. The lateral and medial patellofemoral ligaments support the patellar syntopy. The biceps femoris muscle is located in the lateral region of the section, followed by the caudal crural abductor muscles. In the medial region, the sartorius muscle was visualized cranially. Following the caudal direction of the humerus, we identified the medial head of the gastrocnemius muscle on its caudal surface. The distal femoral caudal arteries and veins are located in the caudal region between the biceps femoris and the semimembranosus muscles. The gracilis extends through the medial region of the section to its caudal apex, where the semitendinosus is located (Figure 3B).

The tibia and fibula constitute the main bone components in the proximal region of the leg as identified on the plastinated image, CT, and MRI of the cranial section. The patellar ligament is visible on the cranial aspect of the tibia. The fibula is positioned caudolateral to the tibia. The cranial tibialis and extensor tendons of the extensor digitorum longus muscle are located lateral to the tibia and contralateral to the sartorius muscle, respectively. The popliteal and flexor digitorum superficialis muscles are located caudal to the tibia. Caudal to the fibula lies the flexor digitorum longus muscle, which is associated with the first digit, followed by the lateral head of the gastrocnemius and biceps femoris muscles, extending laterally across the section. The medial head of the gastrocnemius muscle was also present, followed by the semitendinosus muscle caudomedially, whereas the gracilis muscle extended throughout the medial region of the section (Figure 3C).

In the distal region of the leg, plastinated sections, CT, and MRI revealed that the cranial tibialis muscle was positioned cranially, followed by the extensor digitorum longus and peroneus longus muscles. The tibia was located medially, whereas the fibula was in an intermediate position. Caudal to the fibula lies the flexor digitorum longus, which is associated with the first digit, followed by the flexor digitorum superficialis and the gastrocnemius muscles. The calcaneal tendon is the most caudal structure. The lateral saphenous vein was identified caudolaterally on CT and MRI (Figure 3D).

In the proximal region of the foot, vascularization is observed in the cranial section, represented by the lateral and medial saphenous veins, which belong to the cranial branches. The deepest vein follows the dorsal artery of the foot caudally, with clearer visualization on CT and MRI because of attenuation of the structures. The cranial tibialis and extensor digitorum longus muscles were positioned in the cranial region of the section, followed by the talus and calcaneus. The medial region, from cranial to caudal, includes the medial collateral ligament, medial flexor digitorum, and flexor digitorum lateralis. The lateral region, visualized cranially to caudally, contained the lateral digital extensor muscle, lateral collateral ligament, and the caudally located collateral ligament within the deep digital flexor muscle section (Figure 3E).

In the cranial metatarsal region, as observed in the plastinated CT and MRI sections, the second, third, fourth, and fifth metatarsals were followed by the caudal interosseous muscles. The common dorsal digital vein is located cranial to the metatarsal region. The deep digital flexor muscle is the most cranial section, followed by the superficial digital flexor muscle (Figure 3F). In the distal region of the foot, the medial phalanges of the fourth and fifth digits, proximal phalanx of the third digit, and distal phalanx of the second digit were identified in the plastinated section, CT, and MRI images. The deep digital flexors are caudal to the phalanges. The ungual processes of the distal phalanx were identified in the second and fifth digits. MRI allows identification of the metatarsal pad (Figure 3G).

## 4 Discussion

This study identifies the anatomical structures of the thoracic and pelvic limbs of a wild animal, the crab-eating fox, using a plastination technique to preserve the metameric sections of the animal, correlating its anatomy with CT and MRI. Additionally, this study described the anatomy of the crab-eating fox and how the limb sections correlate with CT and MRI, as well as the evaluation of section shrinkage during the plastination technique. These findings can potentially be extrapolated to other phylogenetically related species, such as the domestic dog, which is more commonly used in veterinary practice and education.

Crab-eating foxes are frequently victims of road traffic accidents (15). Often resulting in immediate death or necessitating urgent medical intervention. In this context, anatomical knowledge is crucial for understanding the species, guiding diagnostic procedures, and supporting clinical decision-making. Previous studies have described similarities between the crab-eating fox and domestic dogs, including the musculature of the forearm (16), joint angle measurements, and the formation of the lumbosacral plexus (17). However, Lorenzão et al. (18) emphasized that anatomical description alone was insufficient to identify muscle origin and insertion or the full distribution of the lumbosacral plexus, highlighting the need for hodological studies. These findings suggest an evolutionary signal among canids, as represented in the mixed plexuses of dogs (19), or even a simple anatomical variability within the studied species unit.

In the thoracic limb, the intrinsic muscles of the scapular and humeral regions are similar to those of domestic dogs and other wild carnivores, differing only in the supraspinatus muscle, which has two muscle bellies in its insertion on the capsule of the humeral joint (20) such findings are possibly due to the species’ adaptation to its habits and behavior. However, this feature could not be clearly identified in the plastinated sections, as the cuts began distal to the muscle’s insertion. While plastinated sections enabled identification of major anatomical structures and their correspondence with domestic species, they posed limitations in determining the exact origins and insertions of certain muscles due to their transverse orientation and sectioning intervals.

Accordingly, in the present study, no anatomical differences were observed, corroborating the similarity described by reported in the literature. Nevertheless, interspecific and individual variations must be considered, since such findings require different approaches, as described by Junior et al. (21) in the sacrocaudal approach during epidural anesthesia or analgesia.

Other studies have focused on surgical approaches in *C. thous*, such as osteosynthesis of the ilium and femur (22) and stabilization of the mandibular symphysis after traumatic disjunction (23) in wild crab-eating foxes hit by vehicles. However, neither brings anatomical comparison studies using diagnostic imaging techniques, and anatomical correlations are scarce in studies using this methodology. Many anatomical regions of *Cerdocyon thous* remain insufficiently studied, which reinforces the need to expand anatomical knowledge of this species. The present study addresses this gap by correlating plastinated limb sections with CT and MRI images, offering valuable anatomical insights. These findings may also be extrapolated to phylogenetically related species, such as the domestic dog, due to their anatomical similarities. This integrated approach enhances the understanding of sectional anatomy and contributes to comparative and educational anatomical studies.

The integrated use of plastination, CT, and MRI is a valuable approach for teaching anatomy, aligning with findings from studies that employed cross-sectional anatomy to enhance student understanding. Corroborating what was described by Ahn et al. (24), with the use of cross-sections for teaching the anatomy of *Naemorhedus caudatus*, resulting in an improvement in students’ understanding of anatomy and being a very useful complementary tool for teaching. Likewise, in the study by Jaber et al. (25) with *Fratercula arctica*, and by Kim et al. (26) with *Triakis scyllium*, which highlight the effectiveness of CT imaging for a comprehensive anatomical study. In this way, the present study on *C. thous* is a multimodal knowledge-enhancing tool, with great potential to assist in learning anatomy through the anatomical description of CT and MRI images together with plastination, as they allow for more precise identification of the sections, improving the understanding for improved comprehension of spatial relationships of structures and their syntopic and topographic correlations in each metamere.

Modern imaging methods, such as virtual necropsy and virtual dissection tools, have enabled the identification of various anatomical structures in crab-eating foxes. According to Kanchan et al. (27), virtopsy necropsy enables the reevaluation, reinterpretation, and repeatability of procedures without destroying the cadavers, which is impossible with traditional necropsies. This technique is also a noninvasive modality that is continually evolving (28).

Therefore, virtual necropsy is an excellent tool for various diagnoses, particularly for the virtual manipulation of bodies containing potentially infectious pathogens, such as those encountered during the COVID-19 pandemic. Additionally, it aids in the dissection of different structures such as knee ligaments in cadavers and serves as an effective tool for preparing surgeons for various procedures (29). Paech et al. (30) reported a 27% improvement in students who had access to three-dimensional virtual dissection, with students reporting that they enjoyed and better understood the anatomy when integrated with three-dimensional tools. Unlike cadavers in traditional classes, which may suffer from wear over time and handling or present variations in quality, plastinated materials are durable, safe for handling, and preserve the spatial relationships between anatomical structures. When combined with 3D reconstructions obtained by CT and MRI and with the images from the exams, they provide a multimodal learning environment aspects that are fundamental for anatomical understanding, as they allow for the comprehension of topography and syntopy, thus improving the understanding of structures within each metamere. This highlights that the simultaneous use of multiple methods enhanced learning.

The integration of different methods and techniques to understand anatomy in diagnostic imaging promotes comprehension and provides a valuable alternative for students. This minimizes errors in image interpretation, report writing, and possible mistakes, resulting in a more accurate diagnosis, as highlighted by Silva et al. (31) and Kawashima et al. (4). From an educational point of view, the present research, with the integration of plastinated sections with CT and MRI images, offers a valuable resource for teaching veterinary anatomy. Similarly, in this study of the limbs of the crab-eating fox, the plastinated metameres allowed the recognition of the syntopic and topographic relationships of the anatomical structures. Compared with the anatomy visible on CT and MRI, this approach led to a clearer spatial understanding and differentiation of anatomical structures owing to the varying attenuations and weightings provided by each imaging technique.

The plastination of crab-eating fox limb metamers contributes to our anatomical knowledge of the species, offering several advantages, as described by Alcázar-Chavez (32). These include durability, ease of handling, preservation of the original tissue characteristics (such as shape, size, and color), as well as odorlessness, and non-toxicity. Plastination is an excellent tool for clinical, pathological, and pathophysiological analyses that enables the identification of anatomical structures in various organisms. These advantages align with the findings of the present study and highlight the benefits provided by plastinated material from the crab-eating fox.

The volume, thickness, and weight of the sections were measured before dehydration and after curing to ensure that the plastination process did not significantly alter the dimensions such as shape, topography, and expected syntropy. According to Brown et al. (33), volume is the best metric to quantify plastination shrinkage. Analysis of the 93 metamers from the crab-eating fox limbs revealed a slight shrinkage of the sections, which did not compromise the identification of the anatomical structures, thus demonstrating the efficiency of the plastination technique and the preservation of details. Tissue characteristics can influence shrinkage to varying degrees, which could explain the minor variation observed in the crab-eating fox limb analysis (34, 35).

Dehydration at varying temperatures is an important factor in tissue shrinkage. Dehydration at room temperature results in greater shrinkage than cold dehydration, leading to minimal shrinkage (33). For the crab-eating fox, low-temperature dehydration was employed, resulting in minimal shrinkage, which did not affect the study objectives or compromise the identification of anatomical structures.

The scarcity of studies that correlate diagnostic imaging techniques, such as CT and MRI, with the anatomy of plastinated specimens creates a significant gap in the literature. However, this study provided unique, innovative, and essential materials that aim to expand our anatomical and imaging knowledge, especially in the field of wild animal studies.

Moreover, these findings enhance our understanding of the anatomical structures of the crab-eating fox, offering valuable educational support to students and veterinary professionals in making informed decisions and selecting appropriate therapeutic approaches for each patient.

## 5 Conclusion

The plastinated metameric sections of the thoracic and pelvic limbs of the crab-eating fox, correlated with CT and MRI, offer a critical anatomical study method for the scientific and academic communities. These sections improve the understanding of anatomy and the interpretation of imaging studies, serving as an educational tool and as a reference to assist veterinary professionals working in anatomy and diagnostic imaging.

The plastinated sections preserved the anatomical features of the specimens, offering advantages such as structural rigidity, identification of structures, absence of odor, and ease of handling, including preservation of the specimen’s anatomy. Despite the associated production costs, which may limit widespread use, their educational and scientific value is significant.

The shrinkage observed in limb volume, weight, and thickness did not compromise the anatomical description or identification, nor did it affect the accuracy of the CT and MRI images, as the variations were negligible. Although this study did not validate the material for applied anatomy or imaging education in a formal instructional setting, the results provide a strong foundation for future investigations aimed at expanding and validating these resources for veterinary training.

## Data Availability

The original contributions presented in the study are included in the article/Supplementary material, further inquiries can be directed to the corresponding author.

## References

[1] Berta A. Cerdocyon thous. Mamm Species. (1982):1–4. doi: 10.2307/3503974

[2] Bueno ADA Motta-Junior JC. Food habits of two syntopic canids, the maned wolf (*Chrysocyon brachyurus*) and the crab-eating fox (*Cerdocyon thous*), in southeastern Brazil. Rev Chil Hist Nat. (2004) 77:5–14. Available at:. doi: 10.4067/s0716-078x2004000100002

[3] Dela Ossa Nadjar O De La Ossa VJ. Fauna Silvestre Atropellada En Dos Vías Principales Que Rodean Los Montes De María, Sucre, Colombia. Rev Colomb Cienc Anim - RECIA. (2013) 5:158. doi: 10.24188/recia.v5.n1.2013.481

[4] Kawashima T Sakai M Hiramatsu K Sato F. Integrated anatomical practice combining cadaver dissection and matched cadaver CT data processing and analysis. Surg Radiol Anat. (2022) 44:335–43. doi: 10.1007/s00276-022-02890-2 35076752

[5] Bortolini Z Matayoshi PM Santos RV Doiche DP Machado VMV Teixeira CR. Casuística dos exames de diagnóstico por imagem na medicina de animais selvagens - 2009 a 2010. Arq Bras Med Vet e Zootec. (2013) 65:1247–52. doi: 10.1590/S0102-09352013000400042

[6] Vansteenkiste DP Lee KCL Lamb CR. Computed tomographic findings in 44 dogs and 10 cats with grass seed foreign bodies. J Small Anim Pract. (2014) 55:579–84. doi: 10.1111/jsap.12278 25291444

[7] von Hagens G Tiedemann K Kriz W. The current potential of plastination. Anat Embryol (Berl). (1987) 175:411–21. doi: 10.1007/BF00309677 3555158

[8] von Horst C von Hagens R Sora C-M Henry RW. History and development of plastination techniques. Anat Histol Embryol. (2019) 48:512–7. doi: 10.1111/ahe.12497 31532015

[9] Ottone NE Cirigliano V Lewicki M Bianchi HF Aja-Guardiola S Algieri RD. Técnica de Plastinación en Ratas de Laboratorio: Un Recurso Alternativo para la Enseñanza, Entrenamiento Quirúrgico y Desarrollo de la Investigación. Int J Morphol. (2014) 32:1430–5. doi: 10.4067/S0717-95022014000400048

[10] Monteiro YF Silva MVF Bittencourt APSV Bittencourt AS. Plastination with low viscosity silicone: strategy for less tissue shrinkage. Brazilian J Med Biol Res. (2022) 55:e11962. doi: 10.1590/1414-431X2022e11962 PMC9296127 35857995

[11] Dejong K Henry RW. Silicone Plastination of biological tissue: cold-temperature technique Biodur© S10/S15 technique and products. J Int Soc Plast. (2007) 22:2–14. doi: 10.56507/zlmj7068

[12] Medixant (2023). RadiAnt DICOM Viewer. 1–93. Available online at: https://www.radiantviewer.com (accessed March 29, 2023).

[13] Fedorov A Beichel R Kalpathy-Cramer J Finet J Fillion-Robin JC Pujol S. 3D slicer as an image computing platform for the quantitative imaging network. Magn Reson Imaging. (2012) 30:1323–41. doi: 10.1016/j.mri.2012.05.001, PMID: 22770690 PMC3466397

[14] World Association of Veterinary Anatomists (WAVA). (2017). Nomina Anatómica veterinaria. 160. Available online at: https://www.wava-amav.org/wava-documents.html (accessed July 9, 2023).

[15] Cunha H. F. Da Moreira F. G. A. Silva Silvania De Sousa. (2010). Roadkill of wild vertebrates along the GO-060 road between Goiânia and Iporá, Goiás state, Brazil. Acta Sci Biol Sci 32:257–263. doi: 10.4025/actascibiolsci.v32i3.4752

[16] Vélez-García JF Patiño-Holguín C Duque-Parra JE. Anatomical variations of the Caudomedial antebrachial muscles in the crab-eating fox (*Cerdocyon thous*). Int J Morphol. (2018) 36:1193–6.

[17] Varjão N. M. Faria M. M. M. D. De Almeida A. E. F. De S., Adami M. Guerra e Silva R. D. Pinto M. Das G. F. (2014). Características anatômicas do plexo Lombossacral de raposinha-do-mato (cerdocyonthous; linaeus, 1706). Rev Educ Contin em Med Veterinária e Zootec do CRMV-SP 12:37

[18] Lorenzão CJ Zimpel AV Novakoski E da Silva AA Martinez-Pereira MA. Comparison of the lumbosacral plexus nerves formation in pampas fox (*Pseudalopex gymnocercus*) and crab-eating fox (*Cerdocyon thous*) in relationship to plexus model in dogs. Anat Rec (Hoboken). (2016) 299:361–9. doi: 10.1002/ar.23304 26692361

[19] Gomez-Amaya SM Ruggieri MRS Arias Serrato SA Massicotte VS Barbe MF. Gross anatomical study of the nerve supply of genitourinary structures in female mongrel hound dogs. Anat Histol Embryol. (2015) 44:118–27. doi: 10.1111/ahe.12116 24730986 PMC4198519

[20] Vélez-García JF Ramírez-Arias JC Duque-Parra JE. Gross anatomy of the intrinsic muscles of the scapular and humeral joint regions in crab-eating fox (*Cerdocyon thous*, Linnaeus 1776). Acta Sci - Biol Sci. (2018) 40. doi: 10.4025/actascibiolsci.v40i1.37861

[21] Junior P. De S Mattos K. De da Cruz de Carvalho Natan Santos A. L. Q. (2014). Topografia da intumescência lombar e do cone medular em *Lycalopex gymnocercus* (G. Fischer, 1814). Rev Bras Ciência Veterinária 21, 173–177. doi: 10.4322/rbcv.2014.380

[22] da Silva BZ dos Santos EAR da Costa PM Goulart MDA Schmitt B Alievi MM. Osteossínteses de ílio e fêmur em cachorro-do-mato (*Cerdocyon thous*). Acta Sci Vet. (2017) 45:6. doi: 10.22456/1679-9216.86090

[23] Zafalon-Silva B Bittencourt Silva L Medina da Costa P de Aquino Goulart M Regina Queiroz Schmidt V Almeida Ruivo do Santos E. Estabilização de sínfise mandibular com cerclagem após disjunção traumática em cachorro-do-mato (*Cerdocyon thous*). Acta Sci Vet. (2018) 46:1–5.

[24] Ahn S Shin W Han Y Bae S Cho C Choi S. Cross-sectional and skeletal anatomy of long-tailed gorals (*Naemorhedus caudatus*) using imaging evaluations. J Vet Sci. (2023) 24:e60. doi: 10.4142/jvs.23076, PMID: 37532303 PMC10404708

[25] Jaber JR Fumero-Hernández M Corbera JA Morales I Amador M Ramírez Zarzosa G. Cross-sectional anatomy and computed tomography of the Coelomic cavity in juvenile Atlantic puffins (Aves, Alcidae, *Fratercula arctica*). Animals. (2023) 13:2933. doi: 10.3390/ani13182933, PMID: 37760335 PMC10525466

[26] Kim SW Yuen AHL Poon CTC Hwang JO Lee CJ Oh M-K. Cross-sectional anatomy, computed tomography, and magnetic resonance imaging of the banded houndshark (*Triakis scyllium*). Sci Rep. (2021) 11:1165. doi: 10.1038/s41598-020-80823-y, PMID: 33441855 PMC7806778

[27] Kanchan T Saraf A Krishan K Misra S. The advantages of virtopsy during the Covid-19 pandemic. Med Leg J. (2020) 88:55–6. doi: 10.1177/0025817220943035 32758010

[28] Dirnhofer R Jackowski C Vock P Potter K Thali MJ. VIRTOPSY: minimally invasive, imaging-guided virtual autopsy. Radiogr a Rev Publ Radiol Soc North Am Inc. (2006) 26:1305–33. doi: 10.1148/rg.265065001 16973767

[29] Shea KG Cannamela PC Dingel AB Fabricant PD Polousky JD Anderson AF. Anatomic dissection and CT imaging of the anterior cruciate and medial collateral ligament footprint anatomy in skeletally immature cadaver knees. J Pediatr Orthop. (2020) 40:e109–14. doi: 10.1097/BPO.0000000000001398, PMID: 31166245

[30] Paech D Giesel FL Unterhinninghofen R Schlemmer H-P Kuner T Doll S. Cadaver-specific CT scans visualized at the dissection table combined with virtual dissection tables improve learning performance in general gross anatomy. Eur Radiol. (2017) 27:2153–60. doi: 10.1007/s00330-016-4554-5 27568182

[31] Silva AC Wellnitz CV Hara AK. Three-dimensional virtual dissection at CT Colonography: unraveling the Colon to search for lesions. Radiographics. (2006) 26:1669–86. doi: 10.1148/rg.266055199 17102043

[32] Alcázar-Chávez I Olmedo-Pérez G Estrada-Dávila MG Tabarez-Rojas A. Use of anatomical models in the teaching of veterinary anatomy as an animal welfare strategy. Rev Educ Técnica. (2021) 5:15–29. doi: 10.35429/jote.2021.15.5.15.29

[33] Brown MA Reed RB Henry RW. Effects of dehydration mediums and temperature on Total dehydration time and tissue shrinkage. J Int Soc Plast. (2002) 17:28–33. doi: 10.56507/xnqm4606

[34] Henry RW. Silicone Plastination of biological tissue: cold-temperature technique North Carolina technique and products. J Int Soc Plast. (2007) 22:15–19. doi: 10.56507/dgzj6845

[35] Sora MC Binder M Matusz P Ples H Sas I. Slice plastination and shrinkage. Mater Plast. (2015) 52:186–9.

