# Supplementary material for: Integration of CT and MRI imaging with plastination for enhanced veterinary anatomy education: a study on the crab-eating fox (Cerdocyon thous)
Source: Front Vet Sci. 2025 Aug 7;12:1600763. doi: 10.3389/fvets.2025.1600763 (PMC12368507; doi:10.3389/fvets.2025.1600763)
Supplement: Supplementary file 1 [file Data_Sheet_1.pdf]

Supplementary Material

Supplementary materials

Three-dimensional reconstruction from volumetric CT scans enabled the identification of anatomical structures and provided a three-dimensional view of the thoracic and pelvic limbs as well as the animals skeleton (Supplementary Videos 1, 2), with the articular regions highlighted in blue (Supplementary Videos 3, 4).

Video 1. Three-dimensional reconstruction from volumetric computed tomography scans of the thoracic limbs, showing the animal's skeletal structure.

Video 2. Three-dimensional reconstruction from volumetric computed tomography scans of the pelvic limbs, showing the animal's skeletal structure.

Video 3. Joint regions of the thoracic limbs highlighted in blue in the volumetric CT three-dimensional reconstruction.

Video 4. Joint regions of the pelvic limbs highlighted in blue in the volumetric CT three-dimensional reconstruction.
